# The Role of Temperature and Humidity on Seasonal Influenza in Tropical Areas: Guatemala, El Salvador and Panama, 2008–2013

**DOI:** 10.1371/journal.pone.0100659

**Published:** 2014-06-23

**Authors:** Radina P. Soebiyanto, Wilfrido Clara, Jorge Jara, Leticia Castillo, Oscar Rene Sorto, Sidia Marinero, María E. Barnett de Antinori, John P. McCracken, Marc-Alain Widdowson, Eduardo Azziz-Baumgartner, Richard K. Kiang

**Affiliations:** 1 Goddard Earth Sciences Technology and Research (GESTAR), Universities Space Research Association, Columbia, Maryland, United States of America; 2 Global Change Data Center, Code 610.2, NASA Goddard Space Flight Center, Greenbelt, Maryland, United States of America; 3 Influenza Program, Centers for Disease Control and Prevention (CDC) Regional Office for Central America Region, Guatemala City, Guatemala; 4 Influenza Unit, Center for Health Studies, Universidad Del Valle de Guatemala, Guatemala City, Guatemala; 5 National Influenza Center, Ministry of Health of Guatemala, Guatemala City, Guatemala; 6 Health Surveillance Division, Ministry of Health of El Salvador, San Salvador, El Salvador; 7 Division of Meteorology, National Environmental Observatories, Ministry of Environment and Natural Resources of El Salvador, San Salvador, El Salvador; 8 National Influenza Center, Gorgas Memorial Institute of Health Studies, Panama City, Panama; 9 Influenza Division, Centers for Disease Control and Prevention (CDC), Atlanta, Georgia, United States of America; Imperial College London, United Kingdom

## Abstract

**Background:**

The role of meteorological factors on influenza transmission in the tropics is less defined than in the temperate regions. We assessed the association between influenza activity and temperature, specific humidity and rainfall in 6 study areas that included 11 departments or provinces within 3 tropical Central American countries: Guatemala, El Salvador and Panama.

**Method/Findings:**

Logistic regression was used to model the weekly proportion of laboratory-confirmed influenza positive samples during 2008 to 2013 (excluding pandemic year 2009). Meteorological data was obtained from the Tropical Rainfall Measuring Mission satellite and the Global Land Data Assimilation System. We found that specific humidity was positively associated with influenza activity in El Salvador (Odds Ratio (OR) and 95% Confidence Interval of 1.18 (1.07–1.31) and 1.32 (1.08–1.63)) and Panama (OR = 1.44 (1.08–1.93) and 1.97 (1.34–2.93)), but negatively associated with influenza activity in Guatemala (OR = 0.72 (0.6–0.86) and 0.79 (0.69–0.91)). Temperature was negatively associated with influenza in El Salvador's west-central departments (OR = 0.80 (0.7–0.91)) whilst rainfall was positively associated with influenza in Guatemala's central departments (OR = 1.05 (1.01–1.09)) and Panama province (OR = 1.10 (1.05–1.14)). In 4 out of the 6 locations, specific humidity had the highest contribution to the model as compared to temperature and rainfall. The model performed best in estimating 2013 influenza activity in Panama and west-central El Salvador departments (correlation coefficients: 0.5–0.9).

**Conclusions/Significance:**

The findings highlighted the association between influenza activity and specific humidity in these 3 tropical countries. Positive association with humidity was found in El Salvador and Panama. Negative association was found in the more subtropical Guatemala, similar to temperate regions. Of all the study locations, Guatemala had annual mean temperature and specific humidity that were lower than the others.

## Introduction

Influenza is estimated to infect approximately 1 billion people each year with 3 to 5 million severe cases and up to 500,000 deaths worldwide [1], [2]. Influenza epidemics typically occur during winter months in temperate regions. In contrast, the timing of influenza epidemics in the tropics varies and often cannot be easily defined because of insufficient surveillance, multiple annual epidemics [3], or continuous influenza activity throughout the year [4]. Several studies have suggested an association between the environment or climate with influenza transmission because of the apparent spatiotemporal variation in influenza spread [5]–[9].

Temperature and relative humidity (RH) have been linked to influenza virus survivability [10]–[12]. A recent study also showed that the stability of the virus outer membrane, which possibly provides protection for the virus during airborne transmission, depends on temperature [13]. In addition, Lowen et al. [8] showed in a laboratory experiment that virus shedding in guinea pigs was significantly longer in low temperatures. Findings on the relationship between influenza transmission and RH were less consistent [14]: Some studies found that aerosolized virus survival decreased as RH increased, while others showed a two-mode relationship. Meanwhile, Shaman and Kohn [9] argued that absolute humidity (AH) influenced influenza virus survival and transmission efficiency more significantly than RH. The associations between influenza and low temperature and humidity have mostly been observed in temperate regions [15], [16]. But in the tropics, where the annual average of temperature and humidity are normally higher than those in the temperate regions, insufficient evidence exists for such a relationship. Nevertheless, the monthly proportion of influenza positive in a few tropical and subtropical countries seemed to be associated with low temperature [3]. In addition, influenza transmission in the tropics often coincides with the rainy season when absolute humidity is typically at its highest [17].

In the tropics, several regions including northeastern Brazil, Philippines and the western part of India, had high influenza activity during the rainy season [18], [19], but others had semi-annual peaks that are not necessarily associated with rainfall [20]. The direct relationship between rainfall and influenza has yet to be established. It is postulated that rainfall leads to crowding which in turn increases the probability for contact, droplet and aerosol transmission. An experimental study [21] showed that contact transmission, unlike aerosol-borne transmission, remained efficient at 30°C. This study also suggested that contact or very close-range transmission may predominate in the tropics, and that more studies are needed to elucidate the transmission route of influenza in the tropics.

In Central America, influenza surveillance data has been limited and the role of environmental and climatic factors on influenza transmission has not been studied. In an effort to comply with the 2005 International Health Regulations, several countries in Central America initiated influenza pandemic preparedness and response plans during 2006. In the same year, countries in Central America also introduced the Generic Protocol for Influenza Surveillance [22] in order to strengthen influenza surveillance [23]. By 2010, this program already showed a significant improvement in influenza surveillance, as evidenced by the expansion of sentinel surveillance networks and the ten-fold increase in the number of samples reported by National Influenza Centers (NIC's) to the World Health Organization Global Influenza Surveillance and Response System [24].

This increased surveillance capacity in the region has provided a better depiction of the respiratory viruses prevalence throughout the year. The countries in Central America now detect respiratory virus in more than 15–20% of samples tested each month – although the periodicity and intensity are different in each country [25]. For example, in Panama, Nicaragua and El Salvador, influenza epidemics occur in a pattern similar to those of southern hemisphere where the epidemics usually occur at mid-year. In other countries such as Costa Rica, Honduras and Guatemala, influenza activity may also occur to a lesser extent during winter months (December to February) [26], although there can be larger variations in Guatemala. In this study, we used data from the improved influenza and other respiratory virus surveillance systems in El Salvador, Guatemala and Panama to explore the association between weekly proportions of surveillance samples tested positive for influenza and temperature, rainfall and specific humidity.

## Materials and Methods

### Study Area

**Figure 1:**
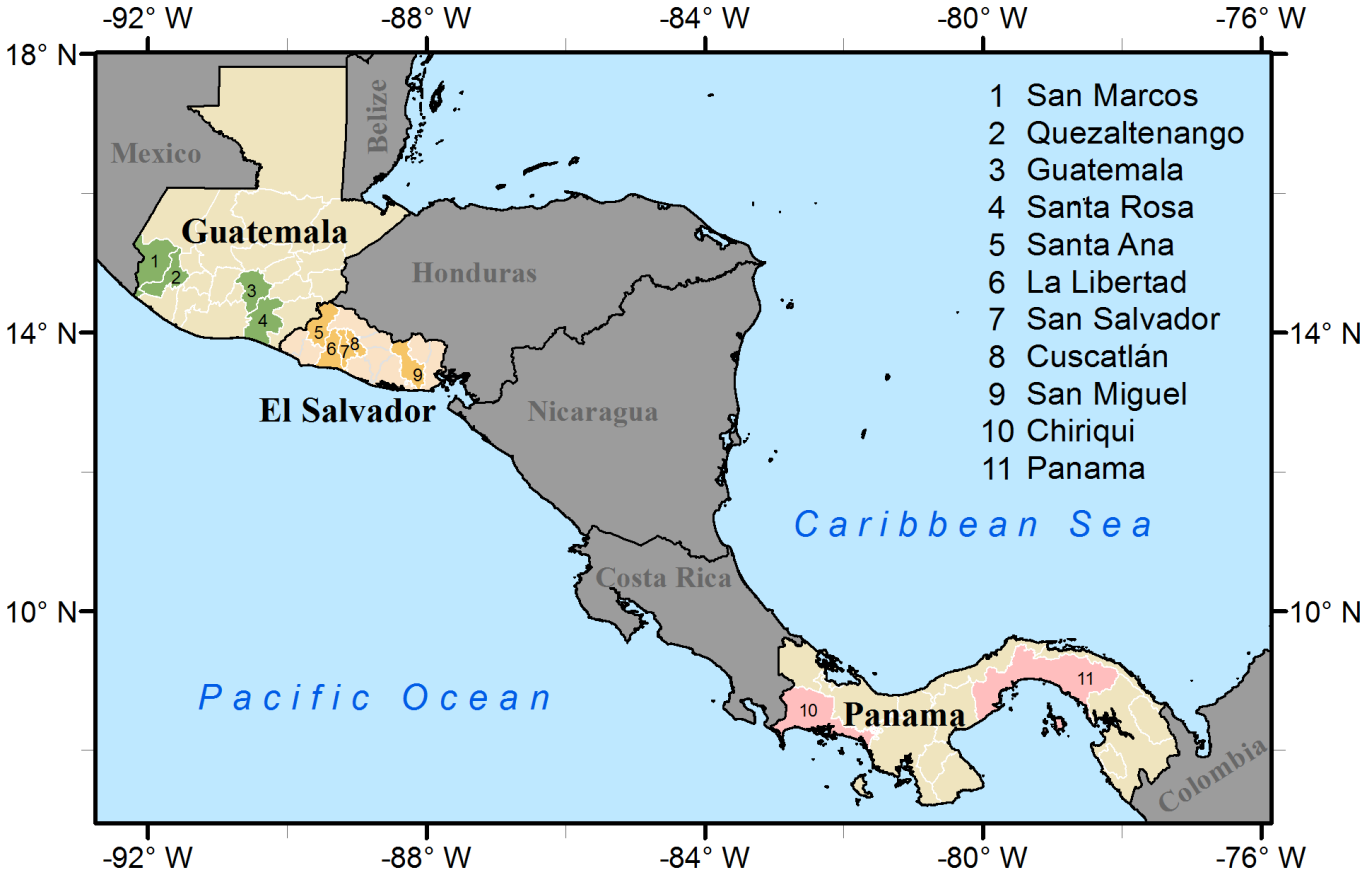
Study areas. Departments or provinces included in the study. Adjacent departments in Guatemala and El Salvador were combined in the analysis: Western departments in Guatemala (1,2), Central departments in Guatemala (3,4) and West-central departments in El Salvador (5–8).

We used influenza surveillance data collected from 11 departments or provinces in 3 Central America countries (Figure 1). These included 4 departments in Guatemala: San Marcos, Quetzaltenango, Guatemala and Santa Rosa; 5 departments in El Salvador: Santa Ana, La Libertad, Cuscatlán, San Salvador and San Miguel; and 2 provinces in Panama: Chiriquí and Panama. To have larger influenza sample sizes, we combined adjacent departments in Guatemala and El Salvador. The combined study areas included western Guatemala departments (San Marcos and Quetzaltenango), central Guatemala departments (Guatemala and Santa Rosa) and west-central El Salvador departments (Santa Ana, La Libertad, San Salvador and Cuscatlán). Other departments or provinces, including San Miguel Department in El Salvador and both Chiriquí and Panama Provinces in Panama – were analyzed individually. Overall, there were 6 study locations.

According to the Köppen climate classification [27] which is based on temperature, precipitation and natural vegetation, these three countries have a tropical wet and dry (savanna) climate. Climate in this zone generally has mean temperature above 18°C year-round and a pronounced dry season. However, there are substantial variations in climate across the region. Most of the cities in Guatemala are located in the mountainous highlands formed by Sierra Madre, the Cuchumatanes range and other sierras. The climate on the highlands is subtropical, and cooler and drier than the rest of Guatemala. Both Panama and El Salvador have less varied topography and are at lower elevation than Guatemala.

### Virological Data

We obtained influenza surveillance data from National Influenza Centers (NIC) at the Gorgas Memorial Institute for Health Studies in Panama [28], the Dr. Max Bloch National Laboratory of the Ministry of Health of El Salvador [29], and the National Laboratory of the Ministry of Public Health of Guatemala [30]. Each NIC compiled and tested respiratory samples from the country's sentinel surveillance system, which is composed of ambulatory clinics and hospitals. There are 9 participating clinics and hospitals in western Guatemala departments, 5 in central Guatemala departments, 13 in west central El Salvador departments, 3 in San Miguel Department in El Salvador, 3 in Chiriquí Province in Panama and 9 in Panama Province [31]. Samples were taken from influenza-like illness (ILI) case-patients in the clinics, and severe acute respiratory infection (SARI) case patients in the hospitals. In all 3 countries, ILI was defined according to WHO criteria [31]: sudden onset of a fever >38°C, either cough or sore throat, and an absence of other diagnoses. SARI case-patient for children under 5 years old was defined as any child <5 years old who was clinically suspected of having pneumonia or severe/very severe pneumonia, and requiring hospitalization [31]. For persons older than 5 years old, SARI was defined as a sudden onset of fever >38°C, either cough or sore throat, shortness of breath or difficulty of breathing, and requiring hospital admissions [31]. Each clinic and hospital selected a convenience sample of case-patient (about 5 samples per week). Influenza was identified using indirect immunofluorescence, and starting in 2009 subtyped through reverse transcription polymerase chain reaction (RT-PCR) [31], [32]. Other respiratory viruses that were also identified using RT-PCR included respiratory syncytial virus (RSV), parainfluenza viruses and adenoviruses. Influenza data in each department or province was collected for at least 3 years, ending in July 2013 (Table 1). In the analysis, we excluded influenza data during the pandemic year period (2009) when influenza transmission was atypical.

**Table 1:** Descriptive statistics for influenza and meteorological data in the study period.

	El Salvador	El Salvador	Guatemala	Guatemala	Panama	Panama
	West-central departments	San Miguel	Central departments	Western departments	Chiriquí	Panama
Departments or provinces included	Santa Ana, Cuscatlán, El Salvador, La Libertad		Guatemala, Santa Rosa	San Marcos, Quetzaltenango		
Study Period	2008–2013	2010–2013	2008–2013	2009–2013	2008–2013	2008–2013
Total samples tested	8395	1169	11270	5053	2130	6841
Influenza positive samples	1591 (18.95%)	113 (9.67%)	2114 (18.76%)	921 (18.23%)	323 (15.16%)	1358 (19.85%)
RSV positive samples	960 (11.44%)	84 (7.19%)	1895 (16.81%)	1059 (20.96%)	227 (10.66%)	731 (10.69%)
Adenovirus positive samples	155 (1.85%)	15 (1.28%)	554 (4.92%)	409 (8.09%)	71 (3.33%)	146 (2.13%)
Parainfluenza positive samples	209 (2.49%)	4 (0.34%)	750 (6.66%)	300 (5.94%)	92 (4.32%)	314 (4.59%)
Temperature (°C)	22.95±1.37	24.75±1.49	20.38±1.40	17.65±1.12	22.83±0.83	25.04±0.59
Specific Humidity (g/kg)	14.29±2.14	14.84±2.32	13.16±1.96	11.63±1.65	15.38±1.47	17.66±1.04
Rainfall (mm/day)	5.09±6.29	4.99±5.61	4.89±5.69	6.59±6.52	9.04±7.45	6.68±6.0

For meteorological data, mean and standard deviation are shown.

### Meteorological Data

The meteorological data for the analysis was obtained from the Tropical Rainfall Measuring Mission (TRMM) satellite and the Global Land Data Assimilation System (GLDAS) [33], [34]. In another study on associating influenza activity in other countries with meteorological variables, we also used meteorological data from ground stations [35]. For the study locations in these 3 countries, however, ground stations were sparsely distributed and their measurements had extensive gaps throughout the study period. Therefore, we did not use ground station data in this study. All meteorological variables in this study were obtained for the same time period as the influenza data.

Rainfall measurements from the Tropical Rainfall Measuring Mission (TRMM) satellite were downloaded via NASA's Goddard Earth Sciences and Data Information Service Center (GES-DISC) Interactive Online Visualization And Analysis Infrastructure (GIOVANNI)[36]. We used the daily precipitation product (TRMM 3B42) with 0.25° by 0.25° spatial resolution (∼25 km at the equator) and a geographical coverage of 50°S–50°N. We averaged all pixels that had more than 10% of its footprint within the study region. Subsequently we took the weekly average in order to match the influenza data temporal resolution.

As a measure for humidity, we obtained specific humidity data. Briefly, specific humidity is the ratio between mass of water vapor and the mass of air (typically expressed in g/kg). It is a similar measure as absolute humidity (please see Supplementary Information for a more detailed description of specific humidity). Near surface specific humidity and temperature for all study locations were obtained from the Global Land Data Assimilation System (GLDAS)[33]. GLDAS is a NASA-NOAA system that utilizes ground and satellite measurements to model global terrestrial geophysical parameters with contiguous spatial and temporal coverage. This dataset also had 0.25° by 0.25° spatial resolution and 3-hourly temporal resolution. Similarly, to obtain the weekly time series for each study region we first averaged the pixels followed by averaging the 3-hourly data into daily data.

All daily meteorological variables were averaged over each week and over two to four previous weeks (i.e. average from the current week to the previous 2 weeks).

### Analysis

Taking into account the influenza surveillance systems in the 3 Central American countries, we calculated the weekly proportion of respiratory samples that were tested positive for influenza virus to represent influenza activity. The commonly used indicator for influenza activity, especially for developed countries in temperate climate zone with established influenza systems, is based on the number of pneumonia and influenza (P&I) mortality, the number of ILI or ARI case patients, or the number of respiratory samples tested positive for influenza viruses. However, such an indicator is not the most suitable for the countries in this study for the following reasons. When using mortality and morbidity data, influenza activity is usually obtained by applying seasonal regression, such as Serfling regression [37]. This approach is not suitable for subtropical countries where influenza activity often does not have a clear seasonal pattern as in the temperate regions. Another estimate of influenza morbidity can also be obtained by multiplying the ILI or SARI cases with the proportion of samples tested positive for influenza. However, the total number of health seeking ILI or SARI cases is not routinely or systematically collected, and therefore not yet part of the surveillance practice in all of the departments or provinces in this study. In the developed countries, large number of ILI or ARI cases are tested for influenza during influenza seasons, hence the number of samples tested positive can, by itself, be used as an influenza indicator. In the three Central American countries in this study, the surveillance systems are nascent and evolving, and only a small proportion of case patients were tested because of the limited throughput of influenza laboratories. Therefore, absolute number of laboratory confirmed influenza cases does not represent the timing of influenza activity well. In the operational setting, influenza positive proportion has been used to determine the influenza timing in Central America. With scant influenza surveillance data available, the proportion of respiratory samples tested positive for influenza (hereafter referred as the “influenza positive proportion”) was considered the most suitable measure to represent influenza activity for the 3 countries in this study. Several influenza studies had also used influenza positive proportion as influenza indicator [3], [38]–[40].

We used logistic regression to model the weekly influenza positive proportion. The logistic regression can model strictly bounded response variable, and is commonly used to describe data on proportions [41]. Other epidemiological studies have used logistic regression to link the disease prevalence with climatic variables [42], [43]. We applied logit function to the influenza positive proportion. Such a function describes a scenario where as the meteorological conditions become more favorable for influenza transmission, more people will be infected, and more specimens will likely be tested positive for influenza. Consequently the odds (logit) for influenza-positive will increase. We performed the logistic regression in R software [44], and we used the methods delineated in [41] to formulate the model for count proportion data where both the influenza positive and negative counts were supplied to the model. More details on this method can be found in the Supplementary Information.

The regression model was fitted individually to each study region using the associated data from the entire study period except for the final year (year 2013), which was reserved for validation. The explanatory variables considered in the regression model were the meteorological variables (temperature, specific humidity and rainfall), positive proportion of other respiratory viruses that co-circulated with influenza (RSV, parainfluenza viruses and adenoviruses), lagged dependent variables (up to lag of 4 weeks), and a polynomial function of the week number (up to degree of 3: week, week^2^, week^3^, where week = 1, 2, 3,… 52). Several studies had found associations between the co-circulating viruses (RSV, parainfluenza virus and adenovirus) and meteorological factors including temperature and rainfall in the tropics [20], [45]. Therefore these viruses were included to adjust for any potential confounding associations between influenza and the meteorological variables. The lagged dependent variable was included since the amount of influenza activity in a particular week depended on the previous week's activity, and also to account for autocorrelation. The week number was included to represent influenza seasonality and other nonlinear relationships that were not represented by the 3 meteorological variables. We first tested the full model as described above. A backward selection (see Text S1 for details) was then applied to select the polynomial order of the week number and the lagged dependent variable, resulting in a reduced model. Autocorrelation was assessed by inspecting the autocorrelation function (ACF) and partial autocorrelation function (PACF) plots. Collinearity among the covariates was assessed by calculating the variance inflation factor (VIF), which is a factor of how much the coefficient's standard error would increase if the said covariate were not correlated with the others. We further tested the full model with different meteorological lags and average periods, resulting in 11 different models. The best model was then selected based on the Akaike's Information Criterion (AIC) (see Text S1 for more details). We did not include interaction terms between predictors because of possible multi-collinearity and lack of clear geophysical interpretations for such terms.

In addition to presenting the Odds Ratio (OR) of the meteorological variable, we also calculated the change in the influenza positive proportion each week when the significant meteorological variables were increased by one standard deviation. We used this measure because it was easier to interpret in terms of the positive proportion rather than the odds for the positive proportion. The change in influenza positive proportion was calculated using meteorological observations throughout the study period. Lastly, to assess the relative contribution of each meteorological variable, we calculated the change in the model deviance when one meteorological variable was removed at a time (more details in Text S1). This change in deviance is a proxy for the relative contribution of each meteorological variable.

A more detailed description of the method can be found in the Supporting Information (Text S1). All statistical analysis was performed using R software [44].

## Results

Influenza data was collected from 2008 to 2013, except for two locations (Table 1): El Salvador's San Miguel Department (2010 to 2013) and Guatemala's western departments (2009 to 2013). During the study periods (excluding the 2009 pandemic year), the proportion of respiratory samples that was tested positive for influenza (influenza positive proportion) in all study locations ranged from 9.67% to 19.85% (Table 1). Similarly, RSV positivity ranged from 7.19% to 20.96%; whereas positivity for adenoviruses and parainfluenza viruses was lower (1.28−8.09% and 0.34−6.66% respectively).

In all locations except for Guatemala, the mean temperature during study period was above 22°C. The mean temperature in Guatemala's western departments was the lowest of all study locations (17.65°C), followed by Guatemala's central departments (20.38°C). Panama Province had the highest mean temperature throughout the study period. Similarly, average specific humidity was the lowest in Guatemala departments, and the highest in Panama. Mean precipitation rate throughout the study period was the highest in Panama (9.04 mm/day in Chiriquí Province, 6.68 mm/day in Panama Province), and the lowest in Guatemala's central departments.

In the analysis, we tested the associations between influenza positive proportion and 3 meteorological inputs (temperature, specific humidity and rainfall), while adjusting for co-circulating viruses (RSV, adenoviruses and parainfluenza viruses), week number, and lagged dependent variables. Eleven models which differed in the meteorological lags and average periods were tested for each study location. The best models (Table 2) were selected based on the AIC. From the best models, we found that influenza positive proportion was significantly associated (p<0.05) with specific humidity in all study locations, whereas significant association with temperature and rainfall were location-specific (Table 2). Specific humidity was positively associated with influenza positivity in west-central departments (Odds Ratio (OR) 1.18, 95% Confidence Interval (CI) 1.07–1.31) and San Miguel (OR 1.32, 95% CI 1.08–1.63) of El Salvador, and Chiriqui Province (OR 1.97, 95% CI 1.34–2.93) and Panama Province (OR 1.44, 95% CI 1.08–1.93) of Panama, but negatively associated with influenza activity in central (OR 0.79, 95% CI 0.69–0.91) and western (OR 0.72, 95% CI 0.60–0.86) departments of Guatemala. On the other hand, rainfall was positively associated only with influenza positivity in Guatemala's central departments (OR 1.05, 95% CI 1.01–1.09) and in Panama Province of Panama (OR 1.10, 95% CI 1.05–1.14). Temperature, however, was only associated with influenza positivity in west-central El Salvador departments, with a 20% reduction in the odds of influenza observed with each degree Celsius increase in temperature (OR 0.80, 95% CI 0.70–0.91). We found that the best model for El Salvador's central departments had meteorological covariates from the previous 1 week, whereas the other locations had meteorological covariates that were averaged over two or more weeks. The resulting polynomial function of the week number for each study location can be found in the Supporting Information (Text S1, Figure S1 and Figure S2).

**Table 2:** Multivariable analysis of meteorological factors associated with influenza positivity.

Country and Province	Adjusted Odds Ratio (95% Confidence Interval)			Meteorological Variable Average Period	Prediction	
	Temperature	Specific Humidity	Rainfall		RMSE	Corr. Coeff
	(°C)	(g/kg)	(mm/day)			
**Guatemala**						
Central departments	1.01 (0.88, 1.15)	**0.79 (0.69, 0.91)**	**1.05 (1.01, 1.09)**	Prev. 1–3 wks ave.	0.08	0.12
Western departments	0.94 (0.80, 1.11)	**0.72 (0.60, 0.86)**	1.01 (0.98, 1.04)	Prev. 0–1 wks ave.	0.13	0.08
**El Salvador**						
West-central departments	**0.80 (0.70, 0.91)**	**1.18 (1.07, 1.31)**	1.00 (0.99, 1.02)	Prev. 1 wk ave.	0.06	0.50
San Miguel	1.28 (0.99, 1.65)	**1.32 (1.08, 1.63)**	0.98 (0.92, 1.05)	Prev. 1–2 wks ave.	0.13	0.02
**Panama**						
Chiriquí	1.30 (0.85, 2.02)	**1.97 (1.34, 2.93)**	0.95 (0.87, 1.04)	Prev. 0–3 wks ave.	0.11	0.73
Panama	1.13 (0.80, 1.61)	**1.44 (1.08, 1.93)**	**1.10 (1.05, 1.14)**	Prev. 1–2 wks ave.	0.07	0.90

Bold font indicates a statistically significant variable (*p-value*<0.05). RMSE is the Root Mean Squared Error and Corr. Coeff is the correlation coefficient between the observation and estimated influenza positive proportion in 2013.

The models were adjusted for: potentially confounding variables (RSV, parainfluenza and adeno viruses), previous weeks' influenza positivity, seasonality and other possible nonlinear relationships (modeled as a polynomial function, up to degree of 3, of the week number).

**Figure 2:**
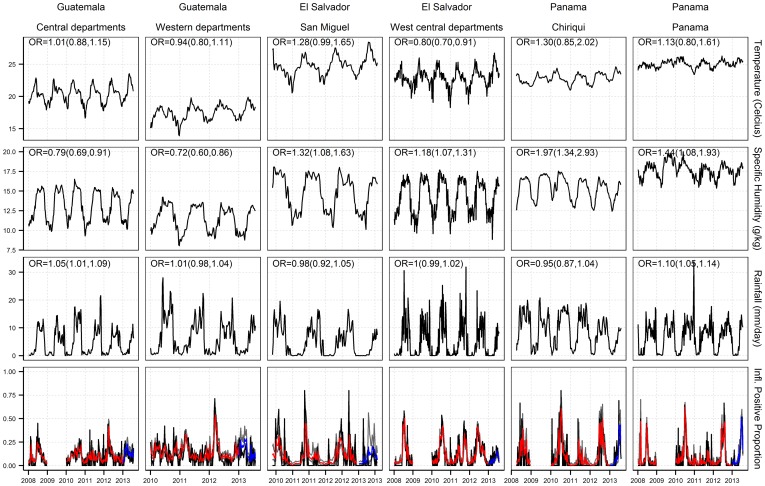
Meteorological parameters, influenza positive proportion and regression output for the study areas. In the last row, black curves are the observed data; grey shades indicate the 95% confidence interval; red curves are modeled results; and blue curves are the prospectively estimated influenza activity using actual meteorological data and regression models trained with influenza data from previous years. OR is the odds ratio from the regression for the meteorological parameters, and CI is the associated 95% Confidence Interval.

The models were subsequently used to estimate influenza positive proportion during the first half of 2013 (January to July 2013) (Figure 2). The blue curves in Figure 2 are the prospectively estimated influenza activity in 2013 using actual meteorological data and regression models trained with influenza data from previous years. The estimated activity closely resembled the actual activity for 4 out of the 6 study areas: Guatemala's central departments, El Salvador's west-central departments, and the 2 Panama provinces. The root mean squared error (RMSE) between the observed and estimated outputs ranged between 0.06 and 0.13, and correlation coefficients between 0.02 and 0.90 (Table 2). Based on the correlation coefficients, the models performed better in El Salvador's west-central departments and Panama provinces, than in Guatemala departments. For Guatemala's central departments, although the correlation coefficient was low, the estimated influenza activity shown in Figure 2 was able to closely follow the actual activity.

In the second best models (with the second lowest AIC), we found that the significant associations between influenza positive proportion and meteorological variables remained the same, except for rainfall in central Guatemala departments (Table S1). Specific humidity was significantly associated with influenza positive proportion in all locations, with inverse relationship in Guatemala and proportional relationship in El Salvador and Panama. Temperature was significant only in El Salvador, and rainfall in Panama Province. In Guatemala's departments and in El Salvador's San Miguel Department, the differences in the AIC values between the best and the second best models were very small (0.36, 0.48 and 0.98 for San Miguel Department, and Guatemala's western and central departments respectively). Typically, as a rule-of-thumb, a difference less than 2 in AIC indicates that the two models are indistinguishable. In Panama Province, the difference in AIC value was 2.07. While larger differences in AIC were found in Panama's Chiriquí Province (3.40) and El Salvador's west-central departments (4.21).

**Figure 3:**
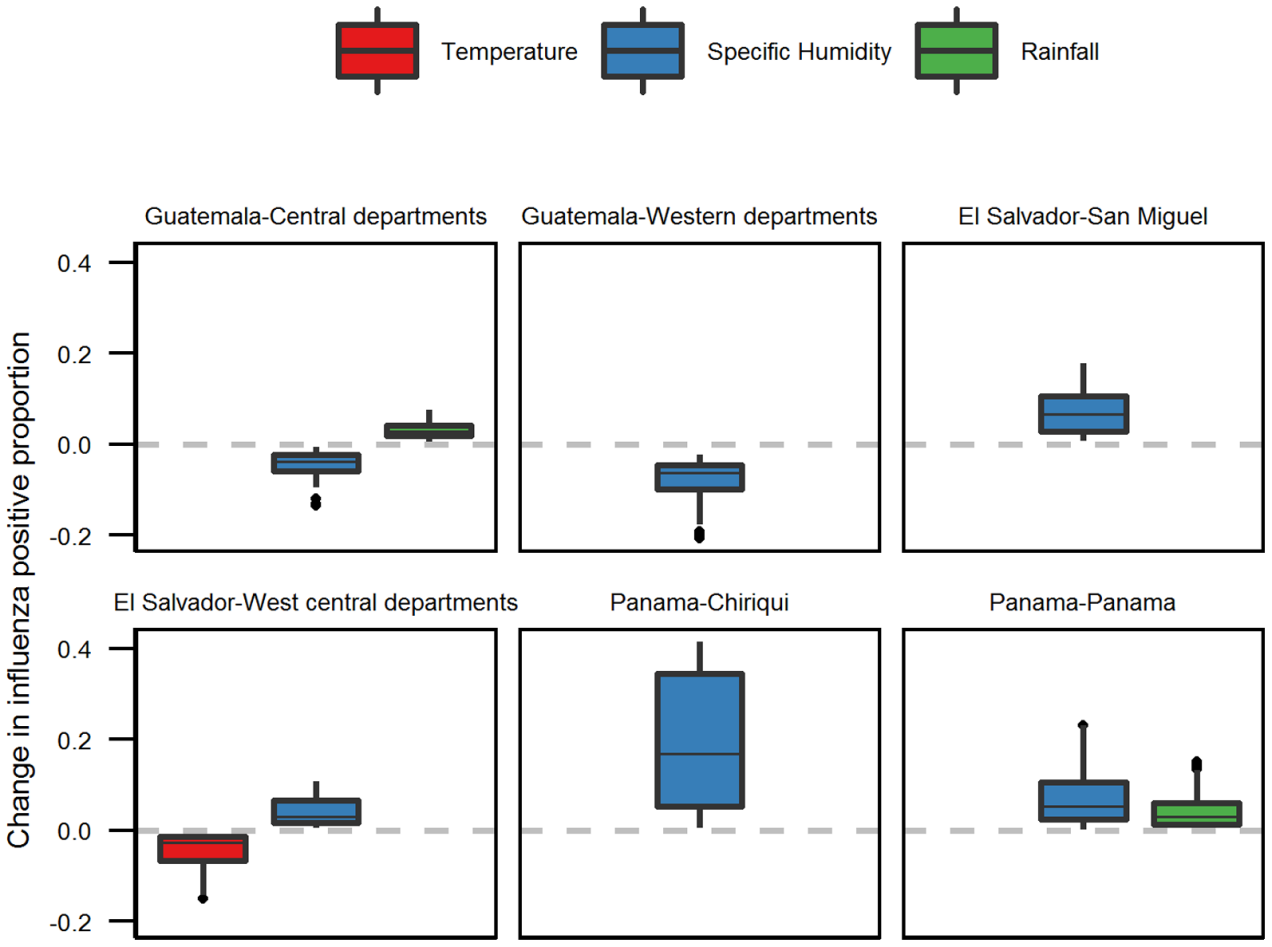
Change in influenza positive proportion when the indicated meteorological variable was increased by 1 standard deviation (temperature 2.74°C, specific humidity 2.61 g/kg, rainfall 6.48 mm/day).

We used the best model to calculate the change in the influenza positive proportion throughout the study period when the significant meteorological variables were increased, one at a time, by one standard deviation (Figure 3). Here, temperature was increased by 2.74°C, specific humidity by 2.61 g/kg, and rainfall by 6.48 mm/day. Overall, the change in the influenza positive proportion was relatively small, ranging from 0.001 to 0.4 (with mean change ranging from 0.03 to 0.2). The mean change in influenza positive proportion when specific humidity was increased by one standard deviation ranged from 0.04 to 0.19. Largest change in positive proportion was found in Panama's Chiriquí Province, and smallest change in Guatemala's central departments. In El Salvador's west-central departments, increases in both temperature and specific humidity resulted in similar change in the influenza positive proportion (−0.01 to −0.14 for temperature, 0.01 to 0.06 for specific humidity). In Panama Province, a one standard deviation increase in specific humidity would result in a slightly higher change in influenza positive proportion as compared to change in rainfall. When either rainfall or specific humidity was increased by one standard deviation, we observed influenza positive proportion change of 0.003 to 0.23 for specific humidity, and 0.001 to 0.15 for rainfall.

**Figure 4:**
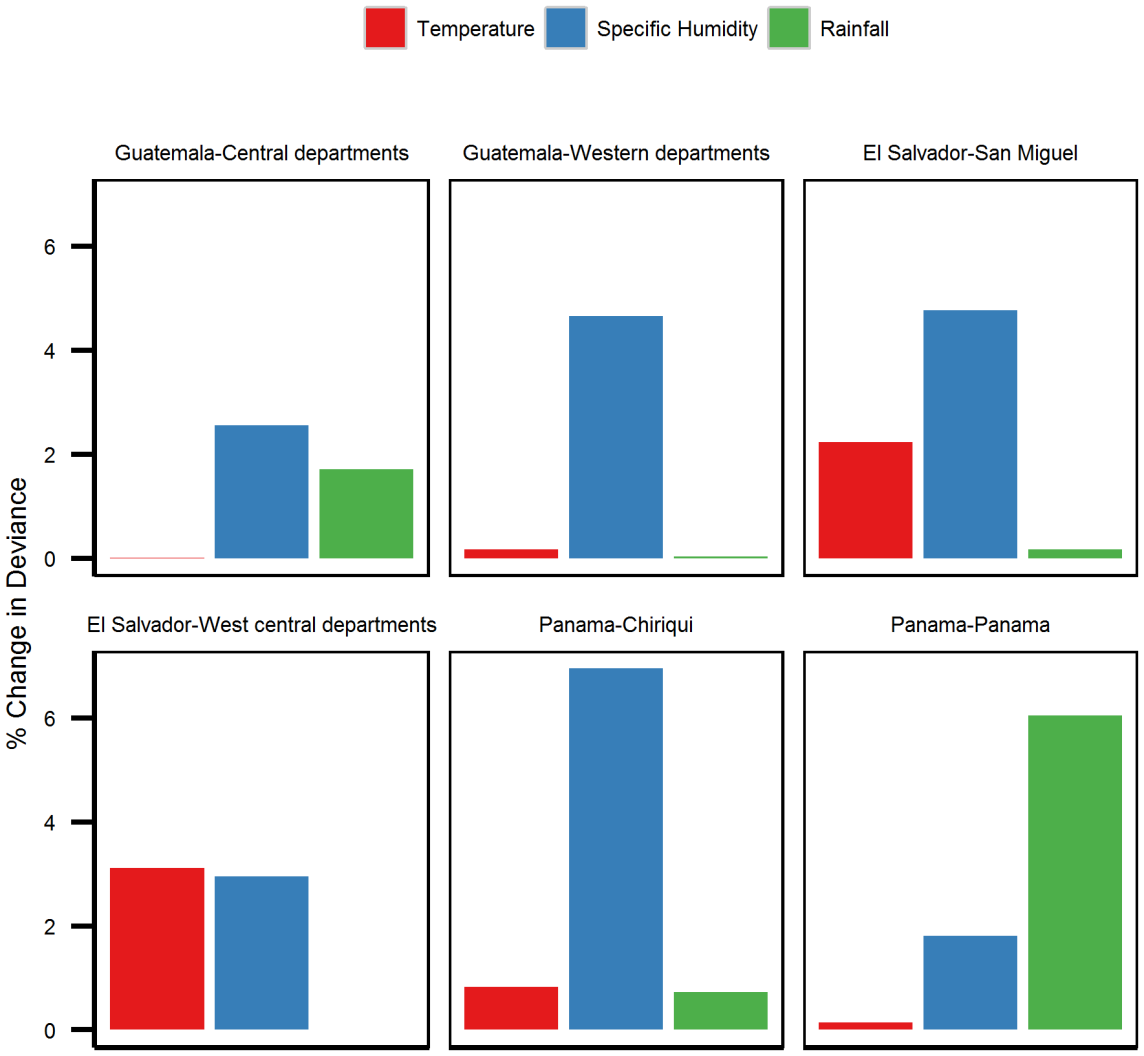
Percent change in model deviance. Change in deviance between the full model (Table 2) and the model with the indicated meteorological variable removed.

From the deviance analysis for the meteorological covariates, we found that the model deviances increased the most (Figure 4) when specific humidity was removed from the models in 4 out of the 6 locations studied (Guatemala's central and western departments, El Salvador's San Miguel Department and Panama's Chiriquí Province). These results indicated that among the meteorological covariates, specific humidity had the highest contribution to the models (4.66% in Guatemala's western provinces, 2.56% in Guatemala's central provinces, 4.77% in El Salvador's San Miguel Department, and 6.95% in Panama's Chiriquí Province). In El Salvador's west-central provinces, both temperature and specific humidity had similar contribution (temperature 3.11%, specific humidity 2.95%). In Panama Province, among the meteorological variables, rainfall had the highest contribution to the model (6.05%) followed by specific humidity (1.81%).

## Discussion

In this study, we evaluated the association between influenza activity – as measured by the proportion of respiratory samples tested positive for influenza (influenza positive proportion) – and meteorological variables in 6 study locations consisting of 11 departments or provinces in 3 Central American countries. After adjusting for previous weeks' influenza activity and other respiratory viruses' activities (RSV, parainfluenza viruses and adenoviruses), we found that specific humidity was significantly associated with influenza activity in all three countries, with proportional relationship in El Salvador and Panama, and inverse relationship in Guatemala. Temperature was found to be significantly and inversely associated with influenza activity in El Salvador's west-central departments. Rainfall was proportionally associated with influenza in Guatemala's central departments and Panama's Panama Province. Among the meteorological covariates, specific humidity had the highest contribution to the model in 4 out of the 6 locations studied. Our results emphasized the association between influenza positive proportions and specific humidity across tropical Central America.

Our finding on the association between influenza activity and specific humidity in Guatemala supports results from experimental studies which demonstrate that low humidity is linked to more efficient aerosol-borne transmission [8] and better virus survival [13]. This association had been largely demonstrated in the temperate regions [3], [15], [16], and in a few subtropical countries, such as Taiwan [46] and Hong Kong [35]. Although these 3 countries lie in the same tropical region in Central America and Guatemala is located next to El Salvador, most Guatemala's cities are situated in highlands and have more temperate climate. Among all the study areas, Guatemala's departments are cooler, with a minimum temperature of 8°C and maximum temperature reaching 29°C. Under such conditions, one could expect that aerosol-borne transmission would possibly become more efficient as humidity decreases [8].

In contrast to findings from temperate regions [15] and our result in Guatemala, our analyses of El Salvador and Panama data suggest that there was a significant association between increasing humidity and influenza transmission at those locations. Situated on highlands, the Guatemala departments have a cooler and less humid climate than the study locations in El Salvador and Panama. Our findings in El Salvador and Panama are different from influenza studies in the temperate regions where influenza was inversely associated with specific humidity [15]. However, our results are consistent with studies of other tropical countries. For example, multivariate analysis from Indian data showed a positive correlation between relative humidity and influenza positive isolates. Similarly, in Dakar, Senegal, influenza activity peaked during periods when humidity, rainfall and temperature were high [47], [48]. A time series study on influenza A incidence in subtropical Hong Kong also showed a positive association between humidity and influenza transmission [49]. Furthermore, a recent study indicated that in locations with high specific humidity and temperature, influenza epidemics were characterized by months with highest humidity and rainfall [50]. The positive association between humidity and influenza activity may be indirect, similar to the crowding effect of rainfall that contributes to increased influenza activity. In modern societies, indoor public places may provide opportunities for crowding when it rains or humidity is high, and thus may enhance contact, aerosol and droplet transmission.

Our study indicated that influenza positive proportion was associated with rainfall only in Guatemala's central departments and in Panama Province. However, in the second best model for Guatemala's central departments (with an AIC indistinguishable from the best model), rainfall was not a significant variable although specific humidity remained significant. This result implied that rainfall may not have as strong association with influenza activity in central Guatemala departments. Rainfall is often associated with influenza activity in the tropical countries, such as Philippines, western part of India [18] and French Guiana [51]. As previously mentioned, the association between rainfall and influenza activity is likely to be indirect. Rainfall may lead to indoor crowding and consequently increase the probability for contact and other modes of transmission. A global study on environmental predictors and influenza epidemics found that rainfall was the best predictor for influenza peaks for locations between 12.5°N–12.5°S [50]. Part of our result supported this finding as Panama Province is located approximately between 8°N–9.5°N, while Guatemala and El Salvador lie between 13°N–18°N. In addition, the deviance analysis indicated that rainfall had highest contribution to the model in Panama Province as compared to the other two meteorological covariates. However, we did not find significant association between influenza positive proportion and rainfall in Panama's Chiriquí Province, which is also located between 8°N–9.5°N.

In El Salvador's west-central departments, we found that influenza positive proportion was also significantly associated with temperature in addition to specific humidity. The inverse association with temperature is similar to what was found in the temperate regions and in an animal study [8]. However, temperature in El Salvador does not go as low as in the temperate regions. At higher temperature, aerosol-borne transmission may not be as efficient [21]. Hence our finding of an inverse association between temperature and influenza activity in El Salvador may not indicate a direct causal relationship between cool temperature and influenza transmission. Temperature in El Salvador may be a proxy for other factors which may facilitate influenza transmission which remain unaccounted for in our regression models.

From the models' deviance analysis, we calculated the relative contribution of the meteorological variables to the model. Our findings indicated that these variables could contribute at most 6.95% to the model (specific humidity in Panama's Chiriquí Province). Similarly, we found that when the meteorological variables were increased by one standard deviation, the influenza positive proportion changed, on average, by 0.2 at most. The small contribution of meteorological variables to influenza modeling was also demonstrated in another study [52], albeit with a different model structure. The study showed that absolute humidity accounted for approximately 3% of the influenza variance in the Netherlands, while most variations were explained by the depletion of susceptible population and between-season effects. In spite of the small contribution of meteorological variable to influenza activity, this and other studies [52], [53] showed that meteorological variables helped forecasting influenza epidemics.

When the models were used to prospectively estimate influenza positive proportion in the first half of 2013, the models performed best in Panama provinces and in the west central El Salvador departments. However, the models performed poorly in the Guatemala departments and El Salvador's San Miguel Department. The models' poor performances in these locations may indicated the dynamics that were not accounted for in the models, such as circulating strains, herd immunity, and socioeconomic factors that are difficult to account for mathematically. It is interesting to note that in the locations where the models performed better (west-central El Salvador departments, Chiriquí Province and Panama Province), influenza activity showed a distinct peak each year, whereas in the other locations there were multiple peaks in a year. Another study [50] indicated that meteorological predictors performed especially poorly in estimating influenza peaks in the middle latitude locations (12.5°N/S to 25° N/S), where Guatemala and El Salvador lie.

There were several limitations to our study. The meteorological data used in this study were outdoor measurements, whereas people in modern society spend much of their time indoors. In these 3 countries, indoor space may also be air-conditioned. However, we could not account for the difference in time spent in air-conditioned environments, and other social and economic parameters which may have a role in affecting the association between influenza activity and meteorological factors. We could only infer associations, but not causality, between influenza activity and temperature, specific humidity and rainfall. Consequently, the associations we found may act only as proxies for factors not considered in this study, as we have previously discussed in the case of El Salvador's west-central departments. In the analysis, we did not account for the role of vaccination which may further confound the association between influenza and the meteorological parameters. During 2012, however, the Vaccine Effectiveness Network in Latin America (known as REVELAC-I by its acronym in Spanish) documented that influenza vaccine coverage was typically low among persons targeted for vaccination (21-41% depending on the target age group, unpublished data). Another limitation to this study was the use of convenience sampling, which may contribute to biased results and difficulties for generalization.

Lastly, we used influenza positive proportion as a proxy of influenza activity, although it was not a direct measure of influenza morbidity or mortality. As we have explained in the method section, considering the nascent and still evolving influenza surveillance systems in the 3 countries, there were scant data alternatives. Therefore, the influenza positive proportion was at the moment the most suitable measure to represent influenza activity. In addition, the positive proportion has been adequate to determine the timing of influenza activity in Central America in an operational setting (Azziz-Baumgartner, personal communication), and also in other studies [3], [38]–[40]. By using the positive proportion, we assumed that the dynamics of influenza virus positive proportion followed the dynamics of influenza morbidity or mortality. This assumption had mostly been corroborated in the temperate and subtropical regions [54] [55]. The influenza positive proportion represented the relative dynamics of influenza activity. Therefore, results from our study cannot be used to interpret the absolute magnitude of influenza activity.

## Conclusion

Our study suggested an association between influenza activity and specific humidity in the tropical Central American countries. Over Guatemala's highlands, where the climate was more subtropical than tropical, and where the mean annual temperature was the lowest compared to El Salvador and Panama, influenza activity increased with decreasing specific humidity. For El Salvador and Panama, which have a hotter and more humid climate than Guatemala, we found that influenza activity was associated with increased specific humidity. This opposite association with humidity in different climates was also discovered in other studies. It is suspected that higher humidity in the tropics may provide uncomfortable conditions for outdoor activities, promote indoor crowding, and increase contact and other modes of transmission. Lower temperature was only significantly associated with influenza activity in El Salvador's west-central departments and more rainfall was associated with increased influenza activity in Panama Province. Such associations with temperature and rainfall were also discovered in other studies. Further studies may incorporate heat index to better understand how temperature and humidity may work together to affect influenza activity. Interpreting the exact mechanisms of the associations between influenza and meteorological parameters is necessarily complex, especially when imperfect surveillance data is paired with meteorological data of finite spatiotemporal resolution, and when socioeconomic data is minimally available. In spite of the limitations, we demonstrated the possibility of forecasting influenza activity using a trained regression model and expected meteorological conditions (from weather or climate forecast). Just like weather forecast, the accuracy of influenza forecast may vary. It is hoped that with further refinement and more suitable meteorological data, such methodology may provide a sufficiently accurate reference point for public health in preparing for and responding to influenza epidemics.

## Acknowledgments

The authors thank the staff of the laboratory influenza of Ministry of Health of Guatemala, Panama and El Salvador; the environmental team of the Ministry of Environment of each country; the PAHO Influenza Group; and the CDC Influenza Division, including Nancy Cox, Joseph Bresee and Ann Moen. We also thank Luis Bonilla for compiling influenza data, Jason Lefler for processing satellite data and Nivaldo Linares-Perez.

## Supporting Information

Figure S1Polynomial function of the week number (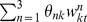 term in Equation 5 of Text S1) for each study location, expressed in term of the dependent variable unit (proportion of influenza positive, 0–1 range). This polynomial term was excluded in El Salvador's San Miguel Department during backward variable selection.(TIF)

Figure S2 Polynomial function of the week number for Panama Province when the secondary outbreaks at the beginning of the year were set to 0 (no influenza activity). Y-axis is in term of the dependent variable unit (proportion of influenza positive, 0–1 range).
(TIF)

Table S1 Odds ratios for the meteorological variables from the 2^nd^ best models.
(DOCX)

Text S1 Details on the meteorological data, methods and the resulting polynomial function of the week number.
(DOC)
