# Supplementary figures and images for: The Role of Temperature and Humidity on Seasonal Influenza in Tropical Areas: Guatemala, El Salvador and Panama, 2008–2013
Source: PLoS One. 2014 Jun 23;9(6):e100659. doi: 10.1371/journal.pone.0100659 (PMC4067338; doi:10.1371/journal.pone.0100659)

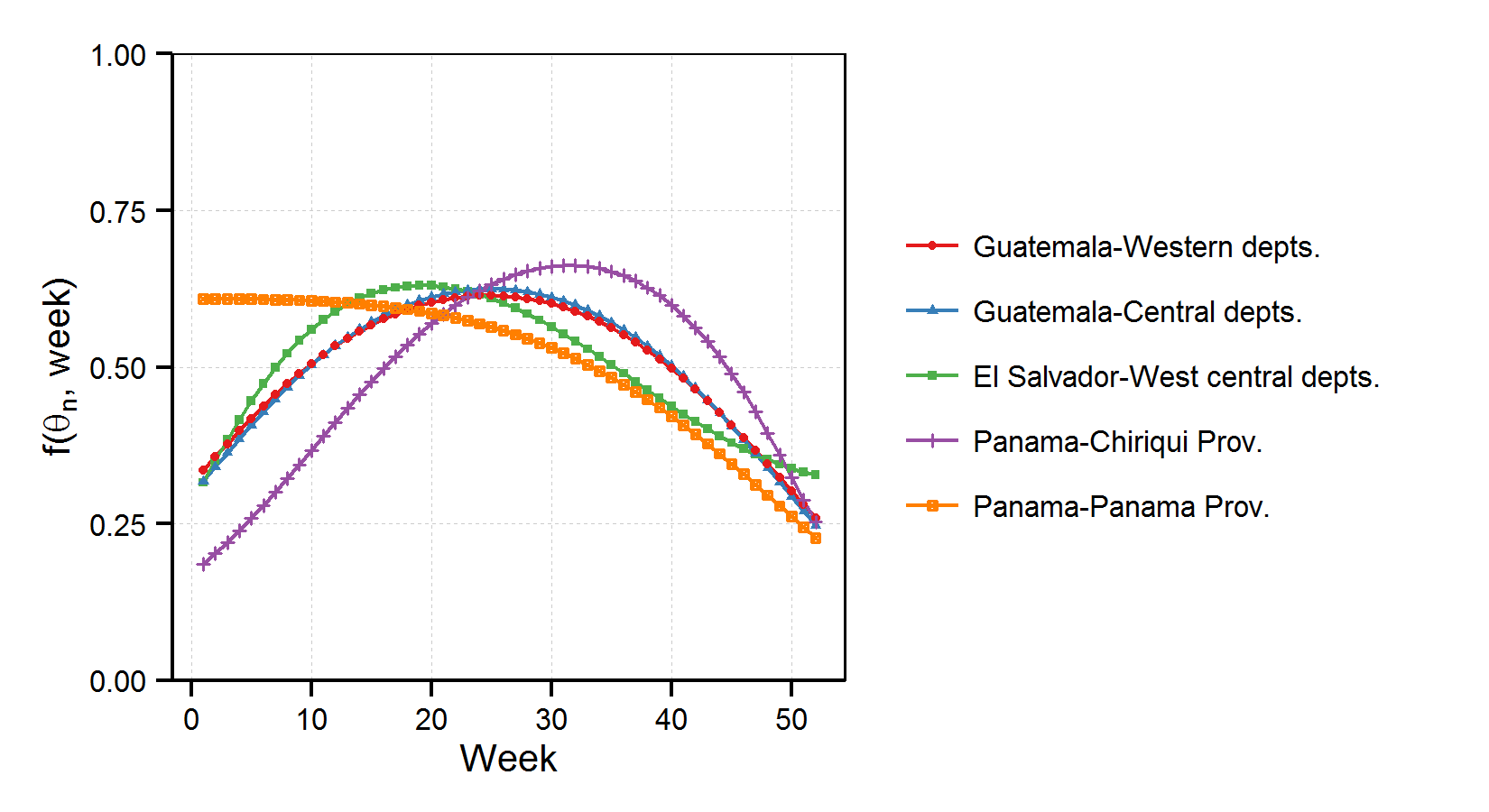

Supplement: Figure S1 — Polynomial function of the week number ( term in Equation 5 of Text S1) for each study location, expressed in term of the dependent variable unit (proportion of influenza positive, 0–1 range). This polynomial term was excluded in El Salvador's San Miguel Department during backward variable selection. (TIF) [file pone.0100659.s001.tif]

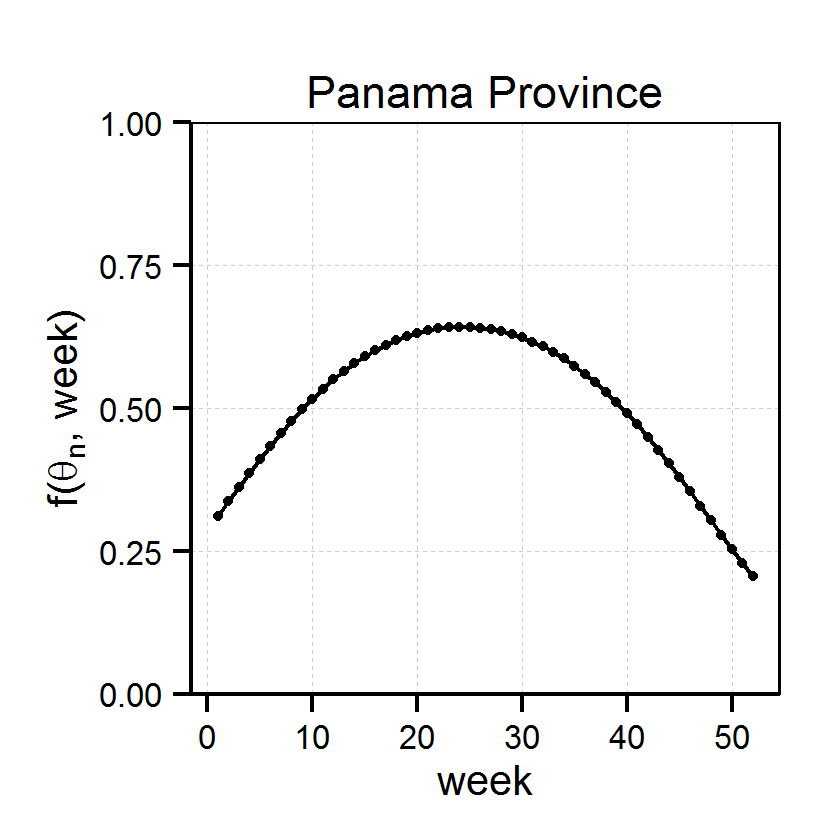

Supplement: Figure S2 — Polynomial function of the week number for Panama Province when the secondary outbreaks at the beginning of the year were set to 0 (no influenza activity). Y-axis is in term of the dependent variable unit (proportion of influenza positive, 0–1 range). (TIF) [file pone.0100659.s002.tif]
