# Supplementary material for: The Role of Temperature and Humidity on Seasonal Influenza in Tropical Areas: Guatemala, El Salvador and Panama, 2008–2013
Source: PLoS One. 2014 Jun 23;9(6):e100659. doi: 10.1371/journal.pone.0100659 (PMC4067338; doi:10.1371/journal.pone.0100659)
Supplement: Table S1 — Odds ratios for the meteorological variables from the 2nd best models. (DOCX) [file pone.0100659.s003.docx]

Table S 1 Odds ratios for the meteorological variables from the 2^nd^ best models. ΔAIC is the difference in AIC between the 2^nd^ best and the best model (AIC_2nd best_ – AIC_best_)

| **Country and Province** | **Adjusted Odds Ratio (95% Confidence Interval)** | | | **Meteorological Variable Average Period** | **AIC** | **Δ AIC** |
| --- | --- | --- | --- | --- | --- | --- |
|  | **Temperature** | **Specific Humidity** | **Rainfall** |  |  |  |
|  | **(°C)** | **(g/kg)** | **(mm/day)** |  |  |  |
| **Guatemala** |  |  |  |  |  |  |
| Central departments | 0.97 (0.86, 1.10) | **0.83 (0.74, 0.94)** | 1.02 (0.99, 1.05) | Prev. 0 to 1 wks ave | 849.21 | 0.98 |
| Western departments | 0.89 (0.75, 1.06) | **0.72 (0.59, 0.87)** | 1.00 (0.97, 1.04) | Prev. 0 to 2 wks ave | 663.44 | 0.48 |
| **El Salvador** |  |  |  |  |  |  |
| West-central departments | **0.80 (0.68, 0.94)** | **1.19 (1.04, 1.35)** | 0.99 (0.98, 1.02) | Prev. 0 to 1 wks ave | 760.98 | 4.21 |
| San Miguel | 1.26 (0.95, 1.66) | **1.32 (1.04, 1.69)** | 0.99 (0.92 ,1.08) | Prev. 0 to 2 wks ave | 270.33 | 0.36 |
| **Panama** |  |  |  |  |  |  |
| Chiriqui | 1.26 (0.86, 1.85) | **1.69 (1.21, 2.40)** | 0.98 (0.93, 1.04) | Prev. 1 to 2 wks ave | 336.05 | 3.40 |
| Panama | 1.24 (0.85, 1.81) | **1.54 (1.12, 2.13)** | **1.10 (1.05 ,1.16)** | Prev. 0 to 2 wks ave | 583.34 | 2.07 |

The models were adjusted for: potentially confounding variables (RSV, parainfluenza and adeno viruses), previous weeks’ influenza positivity, seasonality and other possible nonlinear relationships (modeled as a polynomial function, up to degree of 3, of the week number).
