# Supplementary material for: The Role of Temperature and Humidity on Seasonal Influenza in Tropical Areas: Guatemala, El Salvador and Panama, 2008–2013
Source: PLoS One. 2014 Jun 23;9(6):e100659. doi: 10.1371/journal.pone.0100659 (PMC4067338; doi:10.1371/journal.pone.0100659)
Supplement: Text S1 — Details on the meteorological data, methods and the resulting polynomial function of the week number. (DOC) [file pone.0100659.s004.doc]

## Supplementary Information

### I. Meteorological Data

Precipitation measurements were obtained from the Tropical Rainfall Measuring Mission (TRMM) satellite via NASA Goddard’s Geospatial Interactive Online Visualization and analysis Infrastructure (GIOVANNI) system [1]. We used the daily TRMM3B42 (V7) data product, which was the TRMM-adjusted merged-infrared (IR) precipitation with root-mean-square precipitation error estimates. How precipitation was derived for this data product is beyond the scope of this study, therefore we refer the readers to [2] for details. The spatial resolution of TRMM 3B42 data was 0.25°x0.25° (~ 25x25 km). For each pixel, we calculated the areas covered by the study region. If more than 10% of a pixel area was covered by the study region, then we include the data from the pixel. We then averaged the data across the selected pixels, followed by 7-day average. ArcGIS was used to calculate the pixel area covered by the study region.

We used the Global Land Data Assimilation System (GLDAS) dataset to obtain temperature and specific humidity [3]. This dataset had a spatial resolution of 0.25°x0.25°, and we used the same approach as described for TRMM dataset to select the pixels to be included. Once spatial averaging was performed, we took the weekly average.

As we have previously mentioned, for each meteorological parameter we first took the spatial average to generate one daily time series for each meteorological parameter. We then took the temporal average to create a weekly time series for each meteorological parameter. This weekly time series were also lagged to create 1- to 4-week lag time series. In addition to creating 1-week time series, we also averaged the data over 2 to 4 previous. We created different temporal averages because it was unknown *a priori* which time lag or average was associated with influenza activity. Various temporal aggregates have been used in influenza studies in the literature, from 1-week average to 8 months average [4–7].

**I. 1. *Humidity***

Several humidity measures have been used in influenza studies, including absolute, relative and specific humidity, as well as vapor pressure. In this study, we used specific humidity. Humidity in general is a measure of water content in the air. Briefly, absolute humidity (AH) is the mass of water vapor per unit volume of air; and specific humidity (SH) is the ratio between mass of water vapor and the mass of air (typically expressed in g/kg).

For both AH and SH, as the water vapor content in the air increases, their values also increase. Relative Humidity (RH), on the other hand, depends on temperature. It is a measure of the amount of water vapor in the air compared to the maximum amount of vapor that can exist in the air at its current temperature. Warm air holds more water vapor than cold air. Hence one AH (or SH) can correspond to more than one RH. Relative humidity is defined as the ratio of the partial pressure of water vapor (vapor pressure, E) to the saturated water vapor at the given temperature (E_s_).

In summary, AH, SH and RH can be written as:

$$SH = \frac{m_{v}}{m_{v} + m_{a}}$$

$$AH = \frac{m_{v}}{V}$$

$$RH = \frac{E}{E_{s}}$$

Where,

*m_v_* is the mass of water vapor

*m_a_* is the mass of dry air

*V* is the volume of air

*E* is the partial pressure of water vapor

*E_s_*  is the saturated partial pressure of water vapor

Note that *E_s_* is the partial pressure at which the air can hold the maximum amount of moisture at a given temperature.

I. 2**. *Satellite data validation***

Satellite data validation is beyond the scope of this study. NASA satellite measurements go through stringent calibration and validation processes to ensure satellite data products provide accurate representation of the geophysical parameters (temperature, humidity, radiance, etc.) Some typical calibration and validation efforts are described in calval.jpl.nasa.gov and lpvs.gsfc.nasa.gov.

For example, TRMM data is continuously validated against ground observations located in Darwin (Australia), Houston (Texas), Kwajalein (Republic of the Marshall Islands) and Melbourne (Florida) [8]. The algorithms used to derive the TRMM 3B42 products had been validated at global scale with aggregated gauge data, resulting in correlation coefficient between the two that ranged between 0.545 to 0.864 [9]. Several studies have indicated that the accuracy of TRMM 3B42 varied across regions [10].

It should be noted that satellite-derived measurements are different from ground station observations for the following reasons. Satellite sensors have finite field-of-views. The measurement taken by a satellite sensor represents the average value within a pixel (satellite instrument’s footprint). On the other hands, measurements from ground stations are generally point values. Therefore, satellite measurements are intrinsically different from ground station measurements.

### II. Methods

Logistic regression was used to model the weekly proportion of influenza positive samples. For a site *k* and week *t,* if *Y_kt_* denotes the number of samples tested positive for influenza out of N*_kt_* samples examined, then *Y_kt_* is a binomial random variable. That is, *Y_kt_* ~ *Bin* (*N_kt_*, *p_kt_*) where *p_kt_* is the proportion that are tested positive in site *k* in week *t*. If we denote the logit of the influenza positive proportion as:

$$z_{kt} = \ln\left(\frac{p_{kt}}{1-p_{kt}}\right)$$

the logistic regression is then:

$$z_{kt} = \alpha+ \sum_{j=1}^{3} \beta_{jk} x_{jkt} + \sum_{l=1}^{3} \gamma_{lk} v_{lkt} + \sum_{m=1}^{4} \lambda_{mk} z_{k(t-m)} + \sum_{n=1}^{3} \theta_{nk} w_{kt}^{n}$$

Where,

*x_jkt_* Meteorological variable *j* in location *k* at week *t*; j ϵ {temperature, specific humidity, rainfall}

*v_lkt_* Proportion of samples that are positive for virus *l* in location *k* at week *t* ; l ϵ {respiratory syncytial virus (RSV), Adeno virus, Parainfluenza virus}

*w_kt_^n^* Week number (1 to 52) at location *k*

α Intercept

*β, γ, λ, θ* Regression coefficients

In using the logistic regression model described in equation (4-5), we assumed that the probability of a sample tested positive for influenza followed binomial distribution (i.e. success/failure, or, presence/absence of influenza virus in a sample). Given the meteorological condition in week *t*, the odds for proportion (or probability) of samples that were tested positive would be higher if the meteorological condition was suitable for influenza transmission because more people would likely be infected.

We used R software [11] to perform logistic regression, and supplied both the numbers of specimens tested positive and negative for influenza in each week. This approach for formulating logistic regression is delineated in the textbook by Crawley [12]. Logistic regression can be extended to model population proportion (or probability), where each observation is a proportion based on binomial response variables rather than individual binary scores of 0 or 1.

We fitted the full model as described in equation (5) for each location. A backward selection was then applied to select the polynomial order (denoted as *n* in equation (5)) of the week number (*w*) and the dependent variable lags (denoted as *m* in equation (5)), resulting in a reduced model. The backward selection iteratively eliminates variable whose removal optimizes the model performance. We used Akaike’s Information Criterion (AIC) as a measure for the model performance, which was defined as:

Where for location *k*, Φ_k_ is the number of parameter (regression coefficients: intercept and a coefficient for each independent variable in Equation 5) and _k_ is the likelihood function defined as:

*p_kt_* is the proportion of samples that are tested positive for influenza in location *k* and time *t*, *N_kt_* is the number of samples and *y_kt_* is the number of samples that are tested positive for influenza.

For each location, we tested the model (equation (5)) for the different meteorological lags and average period, resulting in 11 different models. That is, we first tested the model using meteorological variables (*x_jk_*) with 1-week lag, subsequently applied backward variable selection and recorded the AIC values. We then repeated this process using meteorological variables with lag 2 to 4 weeks and meteorological variables that were averaged over 2 to 4 previous weeks. Out of these 11 models, we selected the model with the lowest AIC value as the best model for each location. Lower AIC indicates a better model.

For each model, the severity of collinearity between the meteorological variables was assessed by computing the Variance Inflation Factor (VIF), which is a factor of how much the coefficient’s standard error would increase if the said predictor were not correlated with the others. A value of 1 indicates that the predictor is orthogonal to the others, and common practice considers VIF of 5 or 10 suggests severe collinearity [13,14]. In this study we used VIF of 10 as our threshold. We also assessed the autocorrelation in each model using Autocorrelation function (ACF) and partial autocorrelation function (PACF).

We used the best model to assess how much influenza positive proportion changed when the significant meteorological variables were increased by one standard deviation. For example, we added 2.61g/kg to the observed specific humidity and calculated the influenza positive proportion at each week, while holding the other variables at their observed values. If we denote the resulting influenza positive proportion as $\hat{p}_{1kt}$, and the value of influenza positive proportion when the specific humidity was at their observed value as
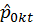
, then the change in positive proportion at time *t* will be:

$$\Delta p_{kt} = \hat{p}_{0kt} - \hat{p}_{1kt}$$

We calculated this change for all data points, *t*, and presented them in a boxplot (Figure 3).

One way to measure the discrepancy between a model and the observed data is using deviance. Model deviance is defined as -2 times the difference in log likelihood of the current model and a saturated model (i.e. one that fits data perfectly) [12]. Smaller deviance indicates a better model fit, analogous to the sum of squared residuals in the ordinary regressions. In R the deviance formula for binomial family is the following [12]:

$$d_{k} = 2 \sum_{t} y_{kt} \ln\left(\frac{p_{kt}}{\hat{p}_{kt}}\right) + (N_{kt}-y_{kt}) \ln\left(\frac{1-p_{kt}}{1-\hat{p}_{_{kt}}}\right)$$

Where *p_kt_* and $\hat{p}_{kt}$are the observed and predicted proportion of influenza positive, respectively; and *N_kt_* is the number of sample.

In order to assess the contribution of each meteorological variable to the model we calculated the percent change in the model’s deviance when the said meteorological variable was removed from the model. Let the deviance of the best model in location *k* be *d_Bk_*. We then removed one meteorological variable from the best model, fitted the reduced model and calculated the deviance, *d_xk_*. The percent change in the deviance will be:

$$\% \mathrm{change} = 100 \times\frac{d_{xk}-d_{Bk}}{d_{Bk}}$$

The increase in the model deviance, when the said meteorological variable was removed, approximately represented the amount of variance for which this variable can account.

### III. Additional Result – Polynomial Function of Week Number

The model used a 3^rd^-order polynomial function of the week number (*w_kt_*) (last term in Equation 5) to represent seasonal and other nonlinear factors not accounted for by the meteorological variables or the other independent variables in the model. Note that we applied a backward selection to the polynomial terms as well as the lagged dependent variable term. With the estimated regression parameter *θ_nk_*, the functions are bounded in all study locations and formed continuous, inverted U-shaped curves (Figure S1) except in Panama Province. For Panama Province, the function started with higher values at the beginning to midyear and fell to lower value toward the end of the year. One possible explanation for the high value at the turn of the year is that the polynomial tried to accommodate secondary influenza outbreaks at the beginning of years 2008 and 2010 (training data) that were not accounted for by the other independent variables in the model. These secondary influenza outbreaks at the beginning of the year could be due to influenza introduced by tourists or returned travelers during the holidays and its subsequent limited propagation. Among these 3 countries, Panama had the highest number of international visitors annually (2010-2012 data) [15]. Furthermore, the tourist’s season that falls between mid-December to mid-April (dry season) coincides with influenza season in the Northern Hemisphere. Hence it is hypothesized that these small outbreaks at the beginning of the year were the limited propagation due to imported influenza cases.

In order to test whether these secondary outbreaks in Panama Province at the beginning of the year caused the jump in the polynomial function, we carried out the analysis with these observed outbreaks set to zero (no influenza activity). The outbreaks were detected using a simple and commonly used approach, by comparing the influenza positivity rate with the annual mean or median value. With these secondary outbreaks excluded, we obtained a continuous, inverted U-shape polynomial for Panama Province (Figure S2) just like in other study locations. Using such setting, the relationships with meteorological variables remained similar to the one reported in the main text (Table 2) where influenza was proportionally associated with both specific humidity (OR = 1.370 (1.003, 1.883)) and rainfall (OR = 1.092 (1.044, 1.141)), and a non-significant relationship with temperature. This test is only a simplified approach to support our postulation for the underlying cause of the polynomial function curve. Further analysis and additional data would be needed to obtain the complete picture.

### IV. Reference
